# Cholic Acid Stimulates MMP-9 in Human Colon Cancer Cells via Activation of MAPK, AP-1, and NF-κB Activity

**DOI:** 10.3390/ijms21103420

**Published:** 2020-05-12

**Authors:** Shinan Li, Trong Thuan Ung, Thi Thinh Nguyen, Dhiraj Kumar Sah, Seon Young Park, Young Do Jung

**Affiliations:** Research Institute of Medical Sciences, Chonnam National University Medical School, Gwangju 501-190, Korea; shinanli@foxmail.com (S.L.); ungtrongthuan@gmail.com (T.T.U.); thinhnt1984@gmail.com (T.T.N.); dhiraj007sah@gmail.com (D.K.S.); drpsy@naver.com (S.Y.P.)

**Keywords:** cholic acid, matrix metalloproteinase-9, reactive oxygen species, AP-1, NF-κB, MAPK, cell invasion, colon cancer cells

## Abstract

Matrix metalloproteinase-9 (MMP-9) plays a crucial role in cell invasion and cancer metastasis. In this study, we showed that cholic acid (CA), a major primary bile acid, can induce MMP-9 expression in colon cancer HT29 and SW620 cells. CA increased reactive oxygen species (ROS) production and also activated phosphorylation of ERK1/2, JNK, and p38 MAPK. Specific inhibitors and mutagenesis studies showed that ERK1/2 and JNK functioned as upstream signals in the activation of AP-1, and p38 MAPK functioned as an upstream signal in the activation of NF-κB. N-acetyl-L-cysteine (NAC, an ROS scavenger) and diphenyleneiodonium chloride (DPI, an NADPH oxidase inhibitor) inhibited CA-induced activation of ERK1/2, JNK, and p38 MAPK, indicating that ROS production by NADPH oxidase could be the furthest upstream signal in MMP-9 expression. Colon cancer cells pretreated with CA showed remarkably enhanced invasiveness. Such enhancement was partially abrogated by MMP-9-neutralizing antibodies. These results demonstrate that CA could induce MMP-9 expression via ROS-dependent ERK1/2, JNK-activated AP-1, and p38-MAPK-activated NF-κB signaling pathways, which in turn stimulate cell invasion in human colon cancer cells.

## 1. Introduction

Colon cancer is the third most common human disease worldwide. The rate of relative survival following diagnosis is 65% at 5 years and 58% at 10 years [1]. Bile acid has been reported to be strongly associated with colon cancer development [2]. However, the molecular mechanisms for the role of bile acid in the development of colon cancer have not been elucidated yet. Bile acid, as the end product of cholesterol catabolism, accounts for a major fraction of daily cholesterol turnover in humans. It plays an important role in the absorption, transport, and metabolism of dietary fats and lipid-soluble vitamins in the intestine [3]. In the duodenum, more than 90% of bile acids are reabsorbed and returned to the liver, which again secretes primary bile acids, cholic acid (CA), and chenodeoxycholic acid (CDCA) [4]. Secondary bile acids deoxycholic acid (DCA) and lithocholic acid (LCA) are formed through bacterial 7α-dehydroxylation of primary bile acids CA and CDCA, respectively [5].

CA, a major primary bile acid, plays an important role not only in the digestion and absorption of dietary lipids but also in cell invasion, growth, and apoptosis through various signaling pathways [6,7,8,9]. NADPH oxidases activated by CA are the major intracellular sources of reactive oxygen species (ROS), which play important roles in modulating signaling pathways, thus changing the cellular phenotype [10,11,12]. Several studies have shown that bile acids can induce ROS production via NADPH oxidase involved in multiple signaling cascades, such as ERK1/2 [13], JNK [14], p38 MAPK [15], and Akt [16].

Cell invasion is a fundamental process for cancer metastasis. It requires increased expression of proteases such as uroplaminogen-type activator (uPA) and matrix metalloproteinases (MMPs) [17]. MMPs are a family of zinc-containing enzymes that are involved in the degradation of different components of the extracellular matrix. There is sufficient evidence indicating that individual MMPs have important roles in tumor cell invasion [18,19]. MMP-9 is involved in cancer metastasis and tumor-induced angiogenesis [20,21]. Furthermore, it has been reported that ROS can activate MAPK (ERK1/2, JNK, and p38 MAPK), which leads to the expression of MMP-9 [22,23]. Some MAPK-activated transcription factors such as NF-κB and AP-1 can regulate the expression of MMP-9 by interacting with the binding site of the promoter of MMP [24].

In colon cell carcinomas, MMP-9 not only serves as a potential prognostic marker of tumor but also an indicator for tumor metastasis [25]. In addition, in a study with T3-T4 node-negative patients, it was found that MMP-9 could be an independent marker of poor prognosis [26]. Therefore, the detailed regulatory relationship between bile acid and MMP-9 should be clarified.

In this study, we demonstrated that CA, a major primary bile acid, can induce cell invasion through MMP-9 expression in human colon cells. We also elucidated the underlying molecular mechanism involved in such induction.

## 2. Results

### 2.1. Induction of MMP-9 by Bile Acids in Colon Cancer Cells

**Figure 1:**
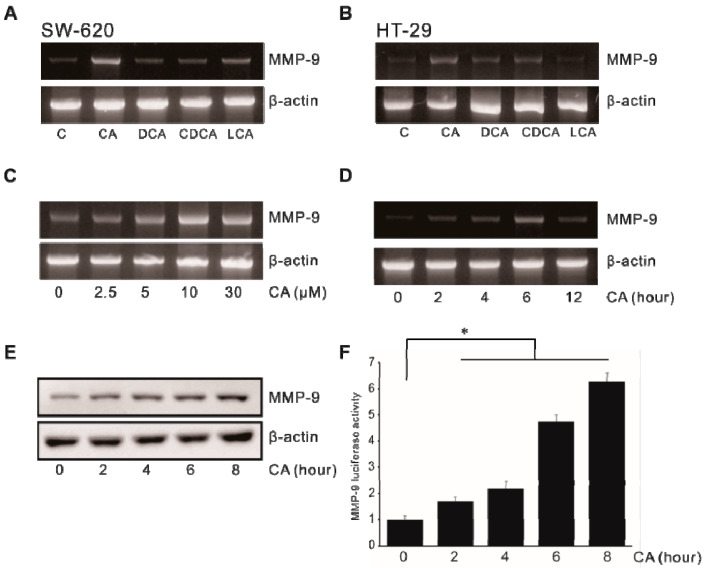
Effect of bile acid on matrix metalloproteinase-9 (MMP-9) expression in colon cancer cells. SW620 cells (**A**) and HT29 cells (**B**) were treated with four different bile acids at 30 μM for 4 h, followed by mRNA extraction and RT-PCR to determine MMP-9 expression level. SW620 cells were treated with cholic acid (CA) at 0–30 μM for 4 h (**C**) or with 10 μM CA for 0–12 h (**D**), followed by mRNA extraction and RT-PCR to determine MMP-9 expression level. (**E**) SW620 cells were treated with 10 μM CA for 0–8 h, and cell lysates were used to determine MMP-9 protein expression using Western blot analysis. (**F**) SW620 cells were transiently transfected with 500 ng pGL4-MMP-9 promoter–reporter construct. These transfected cells were treated with 10 μM CA for 0–8 h and the luciferase activity was determined using a luminometer. Data represent the mean ± standard deviation (SD) from triplicate measurements. * *p* < 0.05 versus control.

To investigate the effect of bile acids on MMP-9 expression, human colon SW620 and HT29 cells were treated with 30 μM of CA, DCA, CDCA, and LCA for 4 h and the level of MMP-9 expression was determined by RT-PCR. As shown in Figure 1A,B, all types of bile acid promoted MMP-9 expression with different levels in both cells (Figure S1). CA and SW620 cells, which most significantly increased MMP-9 among the bile acids and cells used in Figure 1A,B, were employed in the following experiments. To determine the effect of CA on MMP-9 expression, SW620 cells were treated with CA and expression levels of MMP-9 mRNA and protein were measured by RT-PCR and Western blot analysis, respectively. As shown in Figure 1C, CA induced MMP-9 mRNA expression in SW620 cells in a time-dependent manner. The expression of MMP-9 mRNA in cells was increased appreciably after treatment with 10 μM CA. We also found that CA induced MMP-9 mRNA and protein expression levels in a time-dependent manner (Figure 1D,E). To examine the effect of CA on transcriptional regulation of the MMP-9 gene, SW620 cells were transiently transfected with the MMP-9 promoter–luciferase reporter construct (pGL4-MMP-9). The MMP-9 promoter activity was then determined. Cells treated with CA showed an increase in MMP-9 promoter activity in a time-dependent manner (Figure 1F). Consistent with our results, a previous study showed that DCA increases MMP-9 activity, as shown by zymography [27]. Collectively, these results suggest that CA can induce MMP-9 expression in human colon cancer cells.

### 2.2. Involvement of NADPH-Oxidase-Derived ROS in CA-Induced MMP-9 Expression

**Figure 2:**
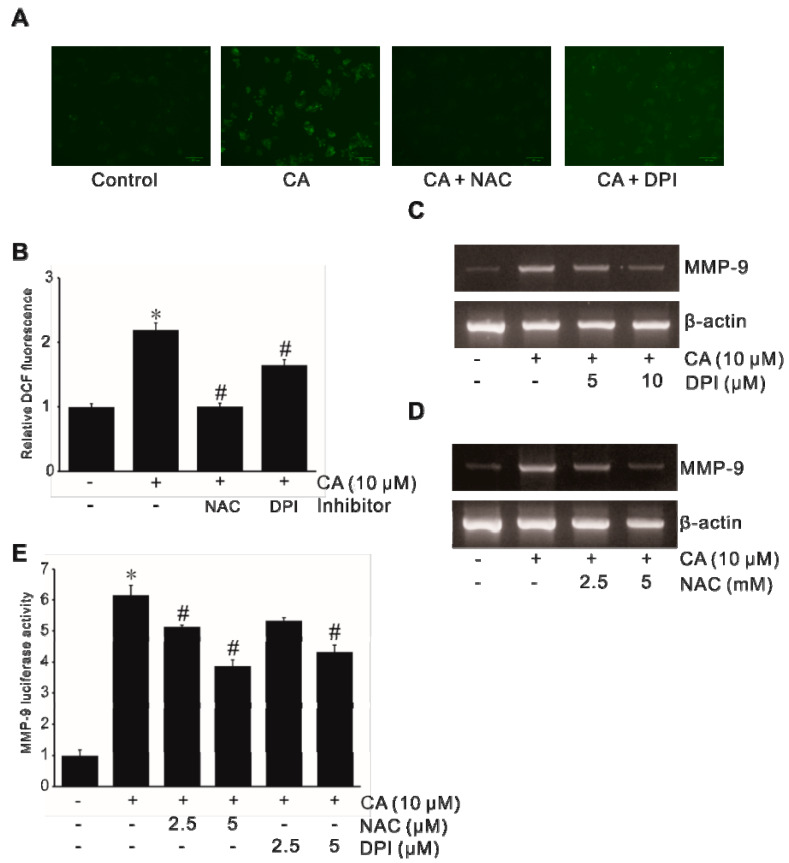
Activation of NADPH-oxidase-derived reactive oxygen species (ROS) during CA-induced MMP-9 expression in colon cancer cells. SW620 cells pretreated with diphenyleneiodonium chloride (DPI) or N-acetyl-L-cysteine (NAC) for 1 h were incubated with 10 μM CA for 10 min. (**A**) Cells were then treated with 5 μg/mL of 5- and 6-carboxyl 2′,7′-dichlorodihydro-fluorescein diacetate (DCFDA) in the dark for 10 min. DCF fluorescence was imaged with a confocal laser scanning fluorescence microscope. (**B**) Statistically significant values of ROS production. Data represent the mean ± standard deviation (SD) from triplicate measurements. * *p* < 0.05 versus control; # *p* < 0.05 versus CA only. SW620 cells pretreated with DPI (**C**) or NAC (**D**) for 1 h were incubated with 10 μM CA for 6 h, followed by mRNA extraction and RT-PCR to determine MMP-9 expression. (**E**) SW620 cells were transiently transfected with 500 ng pGL4-MMP-9 promoter–reporter construct. These transfected cells were pretreated with DPI or NAC for 1 h and then incubated with 10 μM CA for 4 h. The luciferase activity was then determined using a luminometer. Data represent the mean ± standard deviation (SD) from triplicate measurements. * *p* < 0.05 versus control; # *p* < 0.05 versus CA only.

To investigate the effect of CA on ROS generation, SW620 cells were treated with CA and the level of ROS was assayed using the H_2_O_2_-sensitive fluorophore 5- and 6-carboxyl 2′,7′-dichlorodihydro-fluorescein diacetate (DCFDA). As shown in Figure 2A,B, CA induced H_2_O_2_ generation in CA-treated SW620 cells. Such induction was dramatically suppressed by diphenyleneiodonium chloride (DPI, an NADPH oxidase inhibitor) and N-acetyl-L-cysteine (NAC, an ROS scavenger) (Figure S2), indicating that CA might induce ROS generation through NADPH oxidase activation. Furthermore, RT-PCR results showed that CA-induced MMP-9 expression was significantly inhibited by NAC or DPI at the mRNA level (Figure 2C,D). Consistently, similar results were found at the transcription level. As shown in Figure 2E, DPI and NAC inhibited CA-induced MMP-9 promoter activity in SW620 cells. These results confirm that CA can induce ROS generation through NADPH oxidase activation.

### 2.3. Involvement of MAPK in CA-Induced MMP-9 Expression

**Figure 3:**
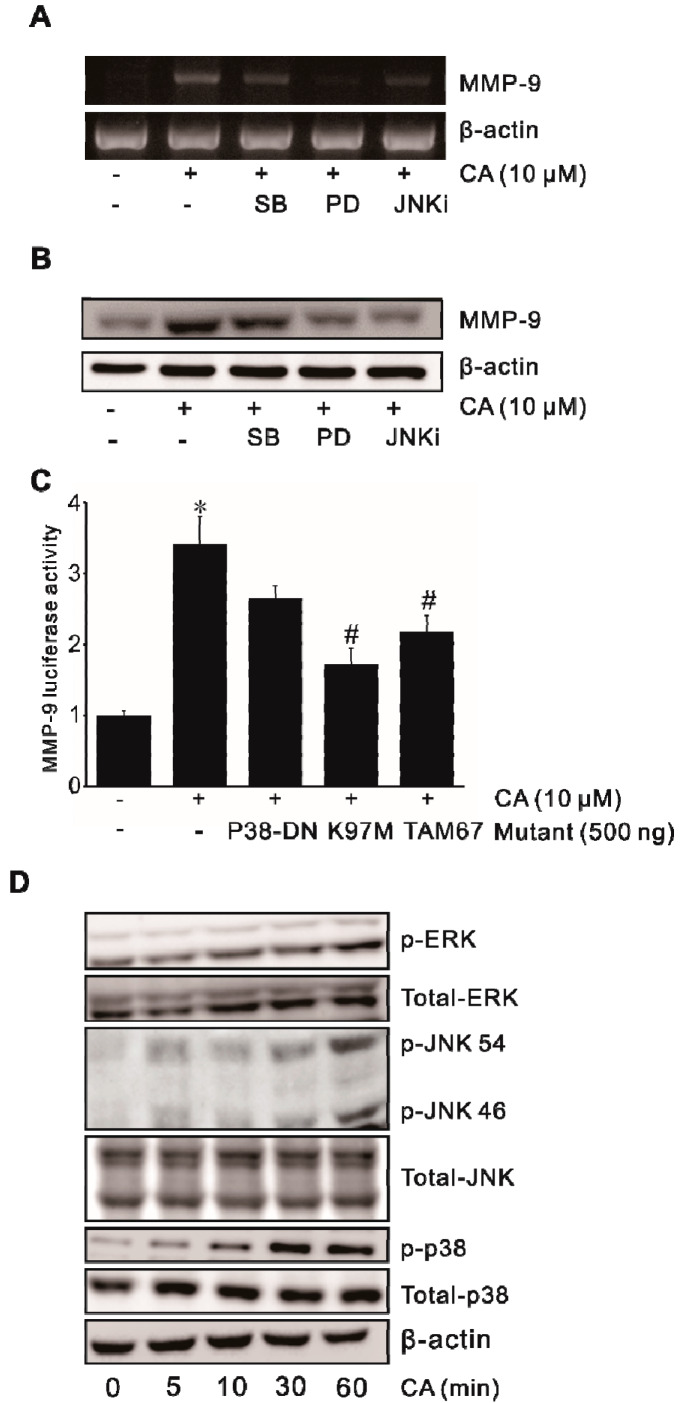
Involvement of MAPK in CA-induced MMP-9 expression. SW620 cells pretreated with 30 μM SB-203580 (SB), 30 μM PD-98059 (PD), and 30 μM JNKi for 1 h were incubated with 10 μM CA for 4 h. Then, MMP-9 mRNA level was measured by RT-PCR (**A**) and protein level was determined by Western blot analysis (**B**). (**C**) SW620 cells were transiently transfected with dominant-negative mutants of MEK-1 (K97 M) or JNK (TAM67), or mutant p38 MAPK (mP38) and co-transfected with PGL4-MMP-9. After incubation with 10 μM CA for 4 h, the luciferase activity was measured using a luminometer. Data represent the mean ± standard deviation (SD) from triplicate measurements. * *p* < 0.05 versus control; # *p* < 0.05 versus CA only. (**D**) SW620 cells were treated with 10 μM CA for 0–60 min, and cell lysates were analyzed using specific antibodies by Western blot analysis.

Our previous studies have demonstrated that MAPK is essential for MMP-9 transcription [20,28]. To explore the mechanism of signaling molecules underlying MMP-9 induction, signaling inhibitors of MAPK (SB-203580, PD-98059, JNKi) were used to determine the molecular mechanisms by which CA induced MMP-9 expression. As shown in Figure 3A,B, inhibitors for ERK1/2, JNK, and p38 MAPK partially blocked CA-induced MMP-9 expression. Consistent with these results, dominant-negative mutant constructs K97M (MEK-1) and TAM67 (JNK), and mutant construct p38 MAPK (p38-DN) significantly inhibited CA-induced MMP-9 promoter activity (Figure 3C). Furthermore, we examined phosphorylation levels of proteins (phospho-ERK1/2, phospho-JNK, phospho-p38 MAPK) of MAPK pathways in SW620 cells by performing Western blot analysis. Phosphorylation levels of these three proteins of MAPK pathways were all increased in a time-dependent manner (Figure 3D), suggesting that the CA-induced MMP-9 expression was mediated through MAPK (ERK1/2, JNK, p38 MAPK) activation in human SW620 colon cancer cells.

### 2.4. Activation of Transcription Factor NF-κB in CA-Induced MMP-9 Expression

**Figure 4:**
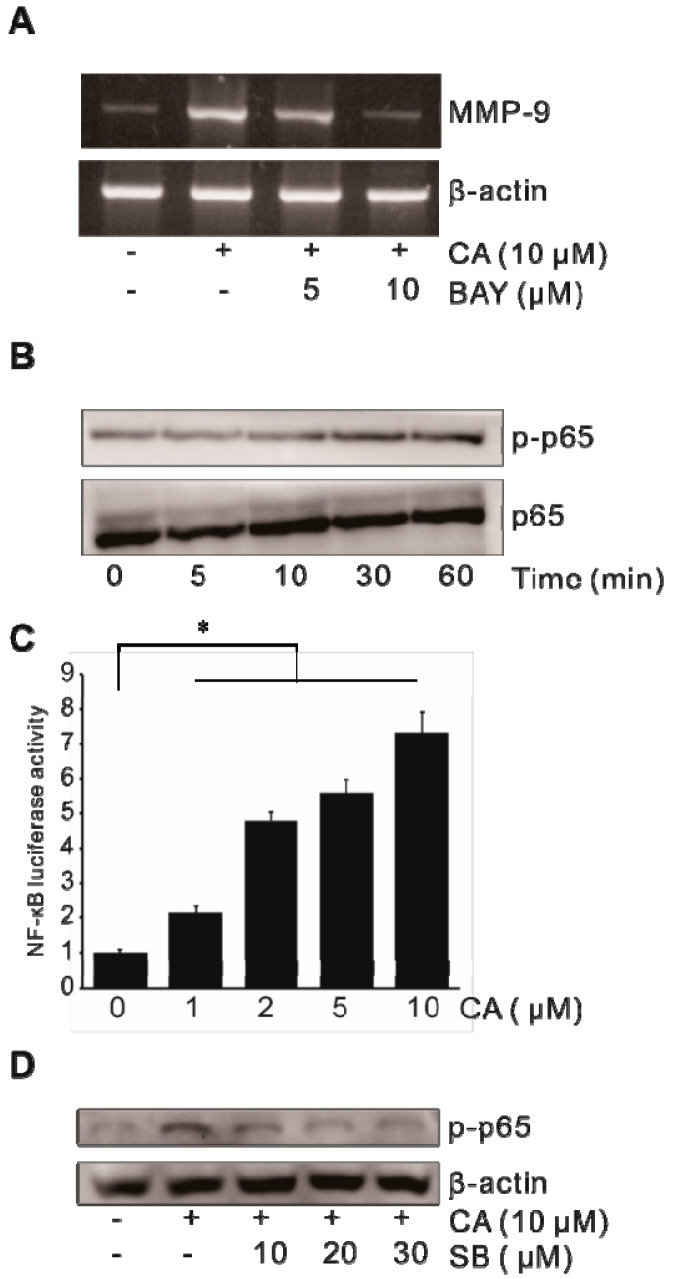
Activation of NF-κB in CA-induced MMP-9 expression in colon cancer cells. (**A**) SW620 cells pretreated with BAY-11-7082 (BAY) for 1 h were incubated with 10 μM CA for 6 h, followed by mRNA extraction and RT-PCR to determine MMP-9 expression. (**B**) SW620 cells were treated with 10 μM CA for 0–60 min and cell lysates were analyzed for total and phosphorylated p65 levels by performing Western blot analysis. (**C**) SW620 cells were transiently transfected with the NF-κB luciferase reporter construct. Transfected cells were treated with 0–10 μM CA for 8 h and the luciferase activity was determined using a luminometer. Data represent the mean ± standard deviation (SD) from triplicate measurements. * *p* < 0.05 versus control. (**D**) SW620 cells pretreated with SB for 1 h were incubated with 10 μM CA for 1 h and cell lysates were analyzed for phosphorylated p65 levels by performing Western blot analysis.

Our previous study showed that transcription factor NF-κB plays an important role in MMP-9 expression [20]. To elucidate the role of transcription factor NF-κB in CA-induced MMP-9 expression, the effect of CA on the activation of NF-κB was investigated in SW620 cells. After SW620 cells were treated with BAY-11-7082 (BAY), an NF-κB inhibitor, CA-induced MMP-9 expression at the mRNA level was examined. As shown in Figure 4A, treatment with BAY resulted in a marked decrease in CA-induced MMP-9 expression. Furthermore, CA enhanced the activation of phosphorylated p65 in a dose-dependent manner (Figure 4B). To examine the effect of CA on the transcriptional regulation of NF-κB, SW620 cells were transiently transfected with an NF-κB promoter–luciferase reporter construct. The NF-κB promoter activity was determined. As shown in Figure 4C, NF-κB promoter activity was increased by CA in a time-dependent manner. To elucidate the role of p38 MAPK in CA-induced NF-κB activation, SB-203580 (SB), a p38 MAPK inhibitor, was used to treat SW620 cells. As shown in Figure 4D, SB blocked CA-induced phospho-p65. These results indicate that NF-κB might be a key molecule in CA-induced MMP-9 expression in SW620 cells.

### 2.5. Activation of Transcription Factor AP-1 in CA-Induced MMP-9 Expression

**Figure 5:**
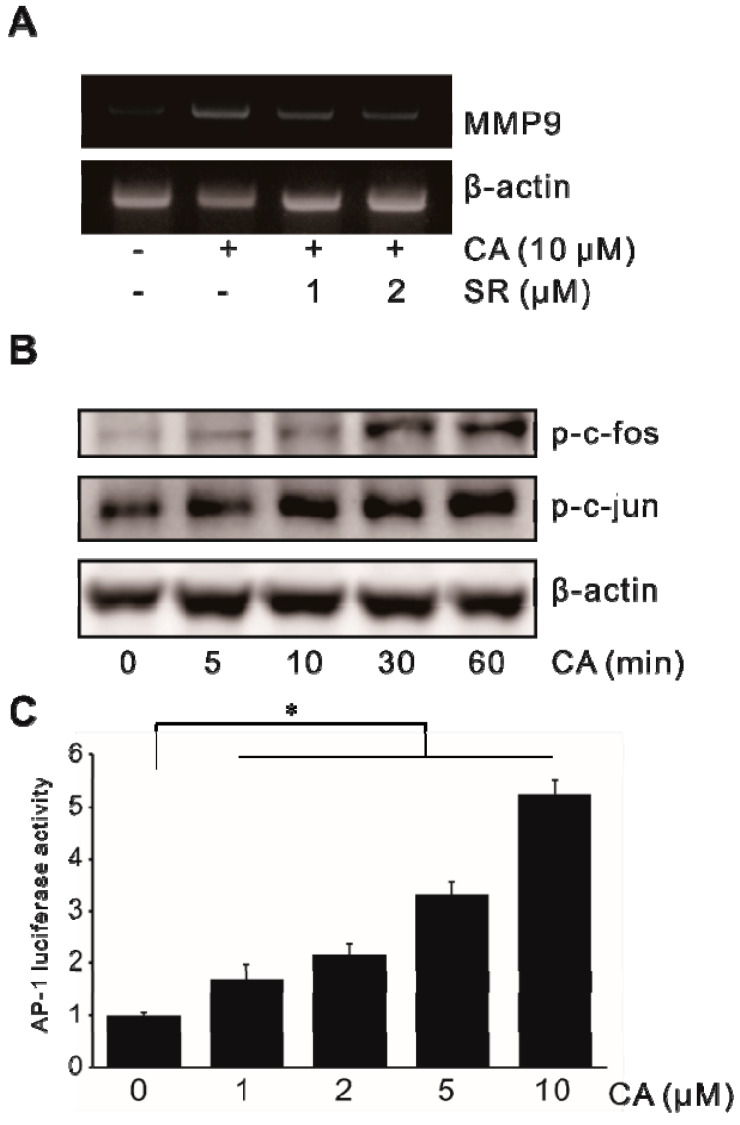
Activation of AP-1 in CA-induced MMP-9 expression in colon cancer cells. (**A**) SW620 cells pretreated with SR-11302 (SR) for 1 h were incubated with 10 μM CA for 6 h, followed by mRNA extraction and RT-PCR to determine MMP-9 expression level. (**B**) SW620 cells were treated with 10 μM CA for 0–60 min and cell lysates were analyzed for phosphorylated c-fos and c-jun levels by Western blot analysis. (**C**) SW620 cells were transiently transfected with the AP-1 luciferase reporter construct. Transfected cells were treated with 0–10 μM CA for 8 h and then luciferase activity was determined using a luminometer. Data represent the mean ± standard deviation (SD) from triplicate measurements. * *p* < 0.05 versus control.

The involvement of transcription factor AP-1 in CA-induced MMP-9 expression was also determined in SW620 cells. To elucidate the role of transcription factor AP-1 in CA-induced MMP-9 expression, SR-11302 (SR), an AP-1 inhibitor, was used to treat SW620 cells. As shown in Figure 5A, treatment with SR decreased CA-induced MMP-9 expression at the mRNA level. CA also induced phosphorylation of c-fos and c-jun (elements of AP-1) (Figure 5B). In the study of AP-1 promoter activity, SW620 cells were transiently transfected with AP-1 promoter–luciferase reporter construct. AP-1 promoter activity was then determined. As shown in Figure 5C, treatment with CA triggered AP-1 promoter activity in a dose-dependent manner. These results suggest that AP-1 could play an important role in CA-induced MMP-9 expression in SW620 cells.

### 2.6. Involvement of Signaling Pathways in ROS-Driven CA-Induced MMP9 Expression

**Figure 6:**
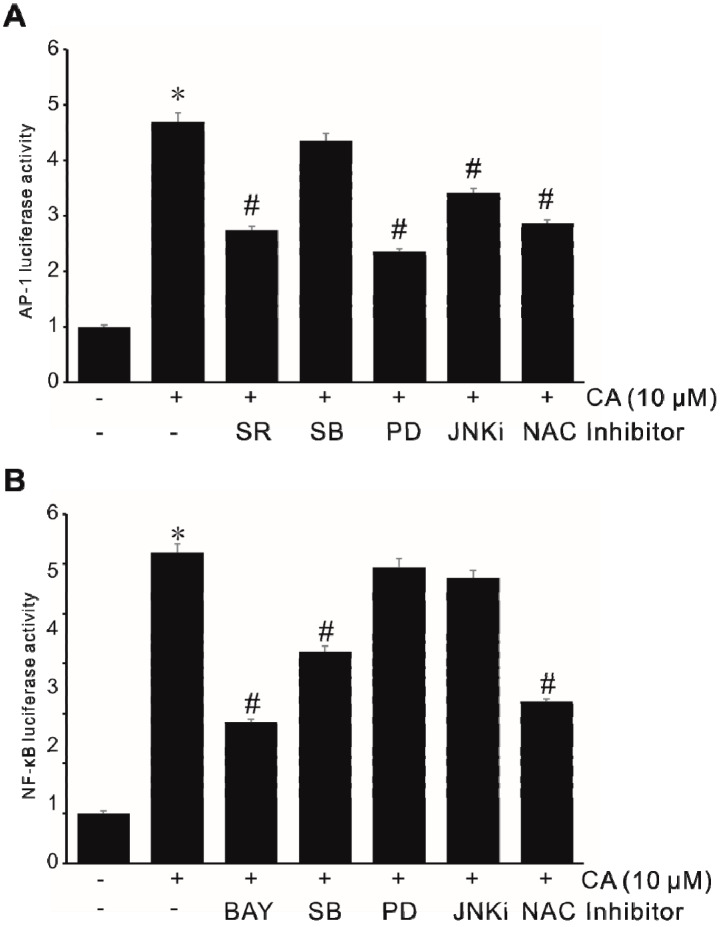
CA-induced AP-1 and NF-κB by ROS generation and MAPK pathway activation in SW620 cells. (**A**) SW620 cells were transiently transfected with the AP-1 luciferase reporter construct. Transfected cells pretreated with 2 μM SR-11302 (SR), 30 μM SB-203580 (SB), 30 μM PD-98059 (PD), and 30 μM JNKi or 5 mM NAC for 1 h were incubated with 10 μM CA for 6 h and then luciferase activity was determined using a luminometer. Data represent the mean ± standard deviation (SD) from triplicate measurements. * *p* < 0.05 versus control; # *p* < 0.05 versus CA only. (**B**) SW620 cells were transiently transfected with the NF-κB luciferase reporter construct. Transfected cells pretreated with 10 μM BAY-11-7082 (BAY), 30 μM SB-203580 (SB), 30 μM PD-98059 (PD), and 30 μM JNKi or 5 mM NAC for 1 h were incubated with 10 μM CA for 6 h and then luciferase activity was determined using a luminometer. Data represent the mean ± standard deviation (SD) from triplicate measurements. * *p* < 0.05 versus control; # *p* < 0.05 versus CA only.

Oxidative species generated via NADPH oxidase in response to bile acids have been implicated in several cellular responses, including the activation of various transcription factors [13,29]. Here, we investigated signaling pathways involved in ROS-driven CA-induced MMP-9 expression in human colon cancer cells. As shown in Figure 6A, AP-1 activities were blocked by inhibitors of ERK1/2 (PD-98059) and JNK (JNKi), whereas the inhibitor of p38 MAPK (SB-203580) did not affect the activity of AP-1. Interestingly, the promoter activity of NF-κB was blocked by the inhibitor of p38 MAPK (SB-203580), whereas the inhibitor of ERK1/2 or JNK did not affect the activity of NF-κB (Figure 6B). These results suggest that CA-induced AP-1 activity is mediated through ROS-dependent ERK1/2 and JNK signaling pathways and that CA induces NF-κB activity through the ROS-dependent p38 MAPK signaling pathway.

### 2.7. CA Promotes Cell Invasion by Stimulating MMP-9 Expression

**Figure 7:**
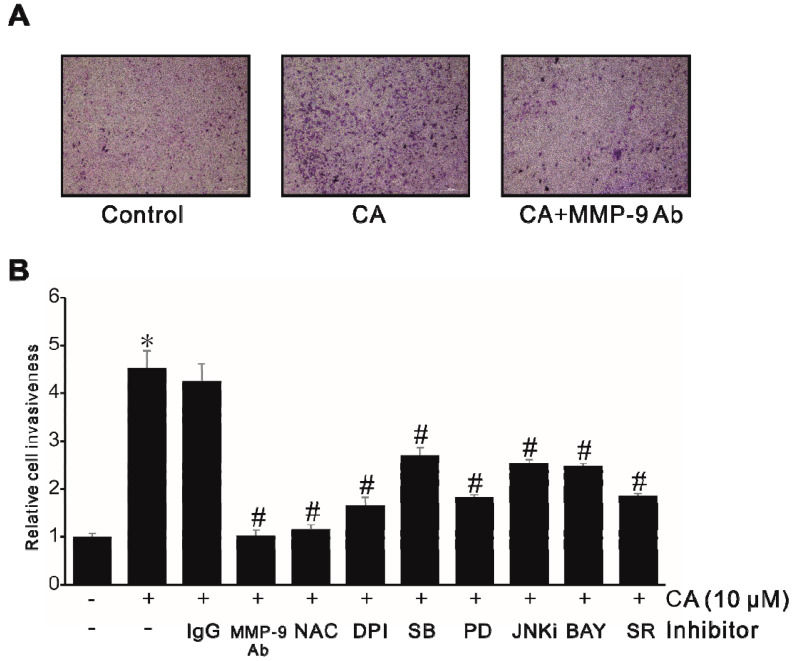
CA-induced cell invasion by stimulating MMP-9 expression in SW620 cells. (**A**) SW620 cells (10^5^) were treated with 10 μM CA in the presence or absence of anti-MMP-9 antibody in a Matrigel apparatus for 24 h. (**B**) SW620 cells (10^5^) were treated with 10 μM CA in the presence of nonspecific IgG (200 ng/mL); anti-MMP-9 antibody; 5 mM NAC; 10 μM DPI; 30 μM SB-203580 (SB); 30 μM PD-98059 (PD); and 30 μM JNKi, 10 μM BAY-11-7082(BAY), or 2 μM SR-11302 (SR) in a BIOCOATTM Matrigel apparatus. After incubation for 24 h, cells invading the undersurface of membranes were stained with a Diff-Quick stain kit and counted under a phase-contrast light microscope. Data represent the mean ± standard deviation (SD) from triplicate measurements. * *p* < 0.05 versus control; # *p* < 0.05 versus CA only (B).

The expression of MMP-9 is essential for the invasive phenotype of cancer cells. The effect of CA on cell invasion was examined by performing a Matrigel invasion assay. As shown in Figure 7A, incubation of SW620 cells with CA resulted in an increased number of invasive cells that passed through the artificial Matrigel. Meanwhile, in the presence of MMP-9 antibody, the number of invasive cells decreased, suggesting that MMP-9 induced by CA might play an important role in colon cancer cell invasiveness. To determine the involvement of ROS, ERK1/2, JNK, p38 MAPK, NF-κB, and AP-1 signals in CA-induced cell invasion, SW620 cells were treated with the indicated inhibitors before treatment with CA. As shown in Figure 7B, inhibitors that blocked CA-induced MMP-9 expression prevented the Matrigel invasiveness of SW620 cells induced by CA. These results suggest that NADPH, ROS, p38 MAPK, ERK1/2, JNK, NF-κB, and AP-1 signals activated by CA could upregulate MMP-9 expression, leading to an increase in colon cancer cell invasion.

## 3. Discussion

The human bile acid pool consists of four different bile acids: two primary bile acids (CA and CDCA) and two secondary bile acids (DCA and LCA) [30]. CA and CDCA are major bile acids in humans [31]. Biliary cholesterol secretion is increased by CA. The amount of cholesterol absorbed was found to be larger with CA (79%) than with CDCA (60%) [32]. Bile acid is involved in the progression of colon cancer. However, many authors are interested in the effect of the secondary bile acid DCA, a proinflammatory and procarcinogenic natural chemical, on bile-acid-sensing receptors such as farnesoid X receptor (FXR) and G-protein-coupled bile acid receptor (TGR5) or gut microbiota study of DCA-induced dysbiosis [33,34,35], while the relevant role of the major bile acid CA in colon cancer progression is ignored. CA, a naturally occurring bile acid, can stimulate cell invasion in human colon cancer cells through activation of multiple signaling pathways [8]. A previous study has shown that CDCA, the primary bile acid, can induce MMP-9 by FAK regulation at the AP-1 motif of the MMP-9 promoter via c-jun activation [36]. Previously, we also reported that bile acids can stimulate invasion of human colon cancer cells [37]. In the present study, we observed that CA treatment could increase colon cancer cell invasiveness and elucidated the molecular mechanisms of CA-induced MMP-9 expression.

ROS, such as superoxide and H_2_O_2_, can act as second messengers in intracellular signaling pathways. They are increasingly involved in cell invasion and migration [38,39]. Previous studies have reported that ROS can act as key regulators in mediating MMP gene expression [40]. AP-1 and NF-κB are involved in the regulation of MMP-9 expression [24]. Bile acids can promote tumor formation on the colon through the generation of ROS [41]. There are several ways that ROS can be produced by the action of bile acids: (i) bile acids can stimulate the release and oxygenation of arachidonate metabolism via cyclooxygenase and lipoxygenase pathways, thus leading to ROS production [42,43]; (ii) protein kinase C activation by bile acids is correlated with the stimulation of reactive oxygen production [44]; (iii) membrane perturbations caused by the hydrophobicity of bile acid can induce ROS production by activating the surface enzyme NADPH oxidase [45]. In our current study, NAC (an ROS scavenger) and DPI (an NADPH oxidase inhibitor) significantly inhibited H_2_O_2_ generation induced by CA, indicating a regulatory role of CA for ROS in MMP-9 expression and cell invasion through NADPH oxidation. 

Invasion and metastases are properties of cancer cells and the final results of a sophisticated series of actions involving multiple signaling molecule interactions [20]. In this study, the blockage of CA-induced cell invasion was observed in SW620 cells with pretreatment of MMP-9 antibody, DPI, or NAC, indicating that ROS production by NADPH oxidase plays an important role in CA-induced MMP-9 expression as well as colon cancer cell invasion. Accumulated evidence shows that ROS production affects invasion and metastases through MAPK signaling pathways [46]. Consistent with our results (Figure 2), in hepatocytes, bile-acid-induced mitochondrial ROS can enhance the downsignaling of ERK1/2 through the ERBB 1-ERKl/2 signaling module [13]. In human breast cancer MCF-7 cells, JNK plays a crucial role in the ROS/MAPK molecular pathway, leading to synthetic lethality upon p53 activation and TrxR inhibition [14]; ROS/MAPK activation by TBBPA-induced NOX plays an important role in MMP-9 expression, and treatment with PD (ERK inhibitor), SP (JNK inhibitor), or SB (p38 MAPK inhibitor) blocked the ROS/MAPK molecular pathways [15]. Transcription factors AP-1 and NF-κB are known to be downstream signals for MAPK [20]. AP-1, a dimeric transcription factor, plays an important role in regulating cell invasion [47], and c-jun and c-fos are two main components of AP-1 [48]. As shown in Figure 5, CA induced both c-fos and c-jun phosphorylation. Consistent with our results, dimerumic acid can suppress H_2_O_2_-induced MMP-7 expression by inhibiting AP-1-mediated gene expression via the JNK/c-jun and ERK/c-fos signaling pathway in SW620 cells [49].

Cross talk and cooperativity between p38 MAPK and NF-κB have been reported [50]. However, the regulation of p38-dependent NF-κB has not been fully elucidated yet. In chondrocytes, COX-2 is expressed via p38 activation/NF-κB recruitment during both differentiation and inflammatory response [51]. Interestingly, it has been reported that mitogen- and stress-activated kinase 1 (MSK1), a potential p38 substrate, can upregulate p65-S276 phosphorylation [52,53]. CA induces phospho-p65 through the activation of p38 MAPK, revealing the regulation of p38 MAPK and NF-κB in human SW620 colon cancer cells.

**Figure 8:**
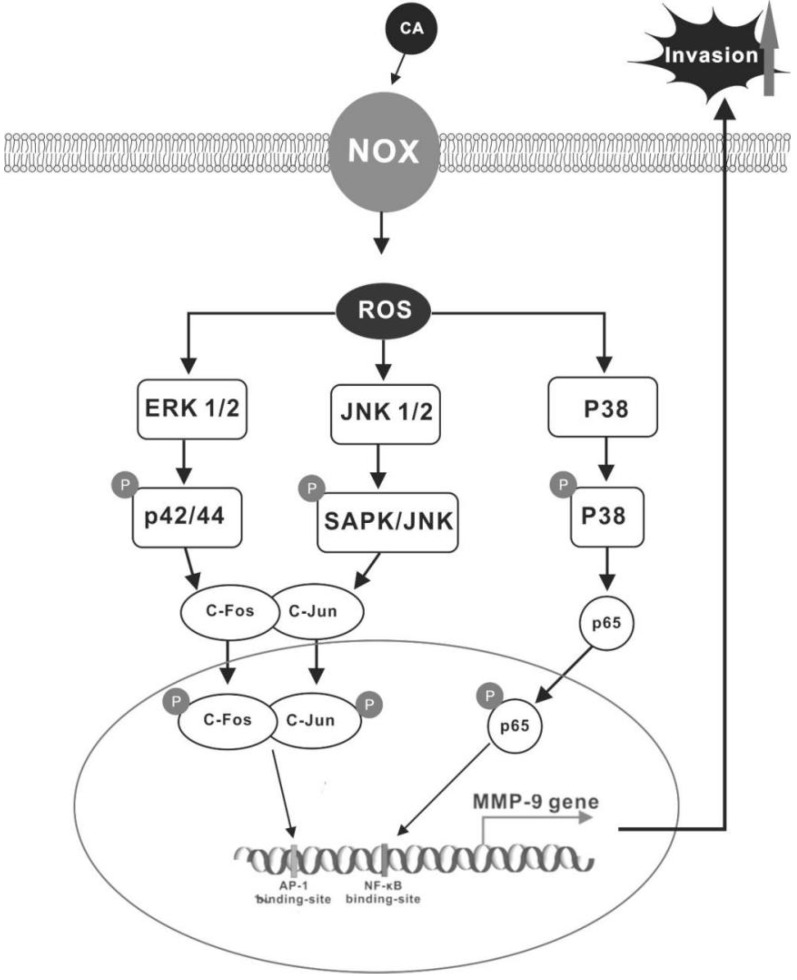
Scheme of the mechanism underlying CA-induced MMP-9 expression in colon cancer cells. CA stimulates NADPH oxidation, which in turn activates ROS-activated MAPK (ERK1/2, JNK, and p38 MAPK). ERK1/2, JNK-activated c-fos/c-jun, and p38-activated p65 then translocate into the nucleus. In the nucleus, phospho-c-fos/c-jun binds to the AP-1 binding site and phospho-p65 binds to the NF-κB binding site, triggering MMP-9 expression and cell invasion.

In conclusion, our results demonstrate that CA can induce MMP-9 expression through ROS-dependent ERK1/2, JNK-activated AP-1, and p38-MAPK-activated NF-κB, thus promoting the invasion of human colon cancer cells (Figure 8).

## 4. Materials and Methods

### 4.1. Cell Culture and Culture Conditions

Human colon cancer cells HT29 and SW620 were obtained from the American Type Culture Collection (Manassas, VA, USA). These cells were cultured in DMEM high-glucose media supplemented with 10% fetal bovine serum (FBS) and 1% penicillin–streptomycin at 37 °C in an atmosphere containing 5% CO_2_. Cholic acid (CA), chenodeoxycholic acid (CDCA), deoxycholic acid (DCA), and lithocholic acid (LCA) were obtained from Sigma Chemical Co. (St. Louis, MO, USA). To determine effects of CA on MMP-9 expression, when cultures reached an appropriate state (3 days), cells were incubated in DMEM containing 0.5% FBS for 12 h at 37 °C and then treated with CA at different concentrations (0–10 μM). The physiological concentration of CA is 10 µM. Levels of MMP-9 messenger RNA (mRNA), protein, and promoter activity were determined. Roles of molecular signaling pathways in CA-induced MMP-9 expression were examined by pretreating the SW620 cells with an MEK inhibitor (PD-98059; New England Biolabs, Beverly, MA, USA), a JNK inhibitor (JNKi; Calbiochem, San Diego, CA, USA), a p38 MAPK inhibitor (SB-203580; Calbiochem, La Jolla, CA, USA), an ROS scavenger (N-acetyl-L-cysteine; Sigma–Aldrich, St. Louis, MO, USA), an NADPH oxidase inhibitor (diphenyleneiodonium chloride; Sigma–Aldrich, St. Louis, MO, USA), an NF-κB inhibitor (BAY-11-7082; Calbiochem, San Diego, CA, USA), or an AP-1 inhibitor (SR-11302; Tocris Bioscience, Ellisville, MO, USA) for 1 h before stimulation with CA.

### 4.2. Reverse Transcription PCR

Total RNA was extracted from cells using TRIzol reagent (Invitrogen, Carlsbad, CA, USA). One microgram of total RNA was used for first-strand complementary DNA (cDNA) synthesis using random primers and M-MLV transcriptase (Promega, Madison, WI, USA). The synthesized cDNA was used as a template for PCR amplification with primer sets for β-actin and MMP-9. Gene-specific primer sequences were as follows: β-actin sense, 5′-AAG CAG GAG TAT GAC GAG TCC G-3′; β-actin antisense, 5′-GCC TTC ATA CAT CTC AAG TTG G-3′ (561 bp); MMP-9 sense, 5′-AAG TGG CAC CAC CAC AAC AT-3′; and MMP-9 antisense, 5′-TTT CCC ATC AGC ATT GCC GT-3′ (516 bp). PCR conditions were as follows: denaturation at 94 °C for 30 s, annealing at 52 °C for 20 s, and extension at 72 °C for 30 s. PCR products were electrophoresed on 1.5% agarose gel containing ethidium bromide.

### 4.3. Western Blot Analysis

SW620 cells treated with CA were washed with phosphate-buffered saline (PBS), detached using Trypsin-EDTA buffer, and stored at -70 °C until use. Proteins were extracted from cells using PRO-PREPTM protein extraction solution (iNtRON Biotechnology, Jungwon, Gyeonggi, KR) with a protease inhibitor mixture (aprotinin, leupeptin, pepstatin A, EDTA, and phenylmethanesulfonyl fluoride) following the manufacturer’s protocol. Fifty micrograms of proteins were then separated by 10% SDS-PAGE and transferred to Immobilon^®^ polyvinylidene fluoride membranes (Millipore Corporation, Billerica, MA, USA). These membranes were blocked with a PBS solution containing 5% nonfat dry milk, incubated with primary antibodies in a blocking solution overnight at 4 °C, and washed three times with TBST (0.1% Tween-20 in TBS) at 10 min intervals. Horseradish-peroxidase-conjugated secondary antibodies (Amersham, Arlington Heights, IL, USA) were used to detect immunoreactive proteins by chemiluminescence. The following antibodies were used: anti-phospho-p44/42 MAPK (ERK1/2) (Cell Signaling Technology, Danvers, MA, USA), anti-phospho-JNK/SAPK (Cell Signaling Technology, Danvers, MA, USA), anti-phospho-p38 MAPK (Cell Signaling Technology, Danvers, MA, USA), anti-MMP-9 (Cell Signaling Technology, Danvers, MA, USA), anti-phospho-NF-κB p65 (Cell Signaling Technology, Danvers, MA, USA), anti-phospho-c-fos (Cell Signaling Technology, Danvers, MA, USA), and anti-phospho-c-jun (Cell Signaling Technology, Danvers, MA, USA). Total protein levels were assayed. After washing the blotted membrane with a stripping solution composed of 100 mM 2-mercaptoethanol, 2% SDS, and 62.5 mM Tris-HCl (pH 6.7) for 30 min at 50 °C, and the membrane was then reprobed with anti-p44/42 MAPK (ERK1/2) (Cell Signaling Technology, Danvers, MA, USA), anti-JNK/SAPK (Cell Signaling Technology, Danvers, MA, USA), p38 MAPK (Cell Signaling Technology, Danvers, MA, USA), anti-NF-κB p65 (Cell Signaling Technology, Danvers, MA, USA), anti-c-fos (Cell Signaling Technology, Danvers, MA, USA), anti-c-jun (Cell Signaling Technology, Danvers, MA, USA), and anti-β-actin (Cell Signaling Technology, Danvers, MA, USA) monoclonal antibodies.

### 4.4. Measurement of Intracellular H_2_O_2_

Intracellular H_2_O_2_ levels were measured using DCFDA (Grand Island, NY, USA) according to a previously described procedure [54]. Briefly, SW620 cells were cultured in DMEM supplemented with 10% FBS until they reached 70% confluence, washed with PBS, and switched to 0.5% FBS DMEM for 2 days. These cells were stabilized in serum-free DMEM without phenol red for at least 30 min before exposure to CA. Cells were then incubated for 10 min with the ROS-sensitive fluorophore H_2_DCFDA (5 μg/mL) and then immediately observed under a laser-scanning confocal microscope. The DCF fluorescence was excited at 488 nm using an argon laser, and the emission was filtered with a 515 nm long-pass filter.

### 4.5. Measurement of MMP-9 Promoter Activity

Plasmid pGL4-MMP-9 promoter (spanning nucleotides from -925 to +13) was kindly provided by Dr. Young-Han Lee (Konkuk University, Korea). SW620 cells were seeded and grown until they reached 70%-80% confluence. The pGL4-MMP-9 promoter plasmid was then transfected into cells using FuGENE 6 (Promega, Madison, WI, USA) according to the manufacturer’s protocol. PRL-TK, an internal control plasmid containing constitutively active Renilla luciferase reporter gene linked to the promoter of herpes simplex thymidine kinase, was transfected as an internal control. Cells were incubated with the transfection medium for 1 day and then treated with CA for 6 h. Co-transfection studies were performed in the presence or absence of the expression vector encoding the dominant-negative mutants MEK-1 (pMCL-K97M), JNK (pMCL-TAM67), or p38 MAPK (pMCL-mP38), kindly provided by Dr. N.G. Ahn (University of Colorado-Boulder, CO), Dr. M.J. Birrer (NCI, Rockville, MD, USA), and Dr. J. Han (Scripps Research Institute, CA, USA), respectively. These cells were harvested with a cell culture lysis reagent (Promega, Madison, WI, USA). Luciferase activities were determined using a luminometer (Centro XS LB960 microplate luminometer, Berthold Technologies, Germany) according to the manufacturer’s protocol.

### 4.6. Transient Transfection of NF-κB and AP-1 Reporter

NF-κB and AP-1 luciferase reporter plasmids were purchased from Clontech (Palo Alto, CA, USA). SW620 cells were washed with Opti-MEM medium and transfected with a pGL-3 vector containing luciferase reporter plasmids using FuGENE 6 (Promega, Madison, WI, USA) according to the manufacturer’s protocol. Reporter-transfected cells were treated with CA for 8 h. The luciferase activity was then measured using a luminometer.

### 4.7. Matrigel Invasion Assay

Cell invasion assay was carried out using a 10-well chemotaxis chamber (Neuro Probe, Gaithersburg, MD, USA) with an 8 μM pore membrane (Neuro Probe) with DMEM containing 10% FBS as the chemoattractant in the lower chamber. SW620 cells (10^5^ in 300 μL) were added to the upper chamber with CA in the presence of MMP-9 antibody or nonspecific IgG and incubated for 24 h so that cells could invade the Matrigel. To determine the effect of signaling inhibitors on CA-induced cell invasion, SW620 cells were preincubated with various ROS scavenger or signaling inhibitors for 1 h and incubated with CA for 24 h. Noninvading cells on the upper surface of each membrane were removed from the chamber, while invading cells on the lower surface of each membrane were stained with a Diff-Quick stain kit (Becton-Dickinson, Franklin Lakes, NJ, USA). After washing with water twice, the chambers were allowed to air-dry. The number of invading cells was counted using a phase-contrast microscope.

### 4.8. Statistics

Data are shown as mean ± SD from at least three separate experiments performed in triplicate. Differences between two datasets were determined by *t* tests. Differences described as significant in the text correspond to *p* < 0.05.

## Supplementary Materials

Supplementary materials can be found at https://www.mdpi.com/1422-0067/21/10/3420/s1.
