# Supplementary material for: Cholic Acid Stimulates MMP-9 in Human Colon Cancer Cells via Activation of MAPK, AP-1, and NF-κB Activity
Source: Int J Mol Sci. 2020 May 12;21(10):3420. doi: 10.3390/ijms21103420 (PMC7279292; doi:10.3390/ijms21103420)
Supplement: Supplementary file 1 [file ijms-21-03420-s001.pdf]

Supplementary Figures

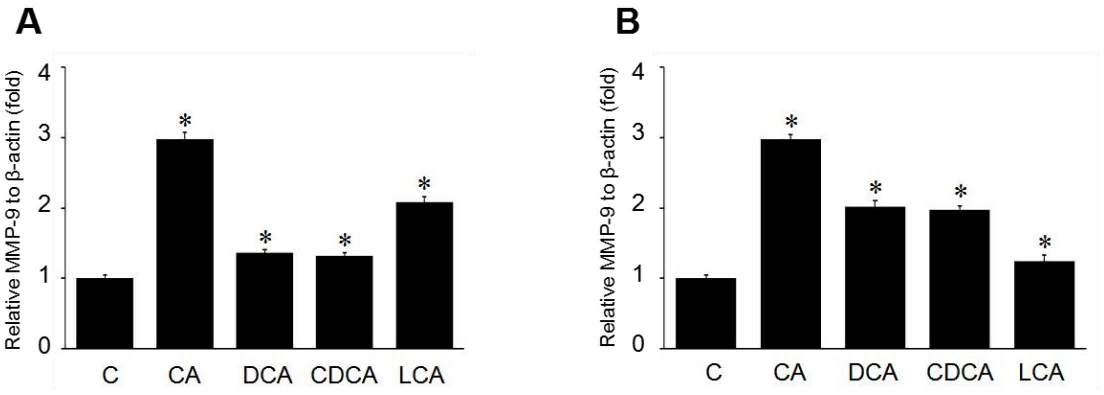

Figure S1. Effect of bile acid on MMP-9 expression in colon cancer cells. SW620 cells (A) and HT29 cells (B) were treated with four different bile acids at 30 μM for 4 h followed by mRNA extraction and RT-PCR to determine MMP-9 expression level. Data represent the mean ± standard deviation (SD) from triplicate measurements. * $P<0.05$ versus control.

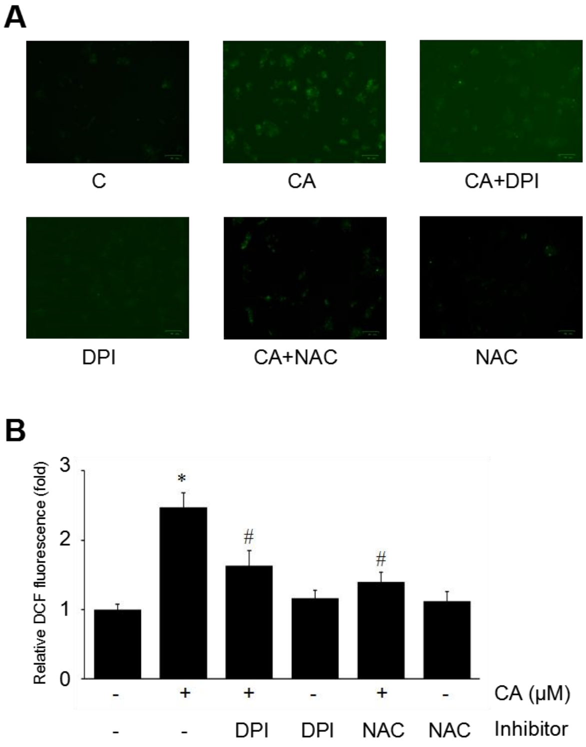

Figure S2. Activation of NADPH oxidase-derived ROS during CA-induced MMP-9 expression in colon cancer cells. SW620 cells pretreated with DPI or NAC for 1 h were incubated with 10 μM CA for 10 min. (A) Cells were then treated with 5 μg/ml DCFDA in the dark for 10 min. DCF fluorescence was imaged with a confocal laser scanning fluorescence microscopy. (B) Statistically significant values of ROS production. Data represent the mean ± standard deviation (SD) from triplicate measurements. * $P<0.05$ versus control; # $P<0.05$ versus CA only.
